# Supplementary material for: COZOID: contact zone identifier for visual analysis of protein-protein interactions
Source: BMC Bioinformatics. 2018 Apr 6;19:125. doi: 10.1186/s12859-018-2113-6 (PMC5889581; doi:10.1186/s12859-018-2113-6)
Supplement: Supplementary file 2 — Software build. Executable binary file of the software tool. (ZIP 118,784 kb) [file 12859_2018_2113_MOESM2_ESM.zip › cozoid/java/docs/beansbinding-1.2.1-doc.zip!/org/jdesktop/beansbinding/PropertyHelper.html]

PropertyHelper (Beans Binding)


---


|  |  |  |
| --- | --- | --- |
| **Overview** \| **Package** \| **Class** \| **Use** \| **Tree** \| **Deprecated** \| **Index** \| **Help** | |  |
| **PREV CLASS**   **NEXT CLASS** | **FRAMES**    **NO FRAMES**     **All Classes** |  |
| SUMMARY: NESTED \| FIELD \| CONSTR \| METHOD | DETAIL: FIELD \| CONSTR \| METHOD |  |


---


## org.jdesktop.beansbinding Class PropertyHelper<S,V>

```
java.lang.Object
  org.jdesktop.beansbinding.Property<S,V>
      org.jdesktop.beansbinding.PropertyHelper<S,V>
```

**Type Parameters:**: `S` - the type of source object that this `Property` operates on: `V` - the type of value that this `Property` represents

**Direct Known Subclasses:**: BeanProperty, ELProperty

---

``` public abstract class PropertyHelper<S,V> extends Property<S,V> ```

An abstract subclass of `Property` that helps with the management of
`PropertyStateListeners` by implementing the methods for adding, removing,
and getting listeners. `PropertyHelper` can be constructed
to manage listeners for multiple source objects, or to ignore the source
object argument when dealing with listeners and associate them directly with
the `PropertyHelper` instance itself. This makes `PropertyHelper`
useful as a base for both property types described in the documentation for
`Property`.

`PropertyHelper` also provides, by way of the protected methods
`listeningStarted(S)` and `listeningStopped(S)` a hook for subclasses
to know when it's time to start tracking changes to a particular source object.

---

**Constructor Summary**

`PropertyHelper()`
  
          Create a `PropertyHelper` that manages listeners for multiple
source objects.

`PropertyHelper(boolean ignoresSource)`
  
          Create a `PropertyHelper`, specifying whether it manages
listeners for multiple source objects, or ignores the source object
argument when dealing with listeners


| **Method Summary** | |
| --- | --- |
| `void` | `addPropertyStateListener(S source, PropertyStateListener listener)`             Adds a `PropertyStateListener` to be notified when the state of the `Property` changes with respect to the given source. |
| `protected  void` | `firePropertyStateChange(PropertyStateEvent pse)`             Notify listeners that the state of this property has changed, as characterized by the given `PropertyStateEvent`. |
| `PropertyStateListener[]` | `getPropertyStateListeners(S source)`             Returns an arry containing the listeners registered for the given source. |
| `abstract  V` | `getValue(S source)`             Returns the value of this `Property` for the given source. |
| `abstract  java.lang.Class<? extends V>` | `getWriteType(S source)`             Returns the type of object that is suitable for setting as the value of this `Property` by calls to `setValue`. |
| `boolean` | `isListening(S source)`             Returns whether or not there are any `PropertyStateListeners` installed for the given source object. |
| `abstract  boolean` | `isReadable(S source)`             Returns whether or not the `Property` is readable for the given source. |
| `abstract  boolean` | `isWriteable(S source)`             Returns whether or not the `Property` is writeable for the given source. |
| `protected  void` | `listeningStarted(S source)`             Called when this `PropertyHelper` changes from having no listeners installed for the given source object to having listeners installed for the given source object. |
| `protected  void` | `listeningStopped(S source)`             Called when this `PropertyHelper` changes from having listeners installed for the given source object to having no listeners installed for the given source object. |
| `void` | `removePropertyStateListener(S source, PropertyStateListener listener)`             Removes a `PropertyStateListener` for the given source. |
| `abstract  void` | `setValue(S source, V value)`             Sets the value of this `Property` for the given source. |

**Methods inherited from class java.lang.Object**

`clone, equals, finalize, getClass, hashCode, notify, notifyAll, toString, wait, wait, wait`

**Constructor Detail**

### PropertyHelper

```
public PropertyHelper()
```

:   Create a `PropertyHelper` that manages listeners for multiple
    source objects.

---


### PropertyHelper

```
public PropertyHelper(boolean ignoresSource)
```

:   Create a `PropertyHelper`, specifying whether it manages
    listeners for multiple source objects, or ignores the source object
    argument when dealing with listeners

    **Parameters:**: `ignoresSource` - whether or not the source argument is ignored when dealing with listeners


**Method Detail**

### getWriteType

```
public abstract java.lang.Class<? extends V> getWriteType(S source)
```

:   Returns the type of object that is suitable for setting as the value
    of this `Property` by calls to `setValue`.

    :   **Specified by:**: `getWriteType` in class `Property<S,V>`
    :   **Parameters:**: `source` - the source object on which to operate **Returns:**: the type of object suitable for setting as the value **Throws:**: `java.lang.UnsupportedOperationException` - if the `Property` is not writeable for the given source **See Also:**: `Property.setValue(S, V)`, `Property.isWriteable(S)`

---


### getValue

```
public abstract V getValue(S source)
```

:   Returns the value of this `Property` for the given source.

    :   **Specified by:**: `getValue` in class `Property<S,V>`
    :   **Parameters:**: `source` - the source object on which to operate **Returns:**: the value of this `Property` for the given source **Throws:**: `java.lang.UnsupportedOperationException` - if the `Property` is not readable for the given source **See Also:**: `Property.isReadable(S)`

---


### setValue

```
public abstract void setValue(S source,
                              V value)
```

:   Sets the value of this `Property` for the given source.

    :   **Specified by:**: `setValue` in class `Property<S,V>`
    :   **Parameters:**: `source` - the source object on which to operate: `value` - the new value for the `Property` **Throws:**: `java.lang.UnsupportedOperationException` - if the `Property` is not writeable for the given source **See Also:**: `Property.isWriteable(S)`, `Property.getWriteType(S)`

---


### isReadable

```
public abstract boolean isReadable(S source)
```

:   Returns whether or not the `Property` is readable for the given source.

    :   **Specified by:**: `isReadable` in class `Property<S,V>`
    :   **Parameters:**: `source` - the source object on which to operate **Returns:**: whether or not the `Property` is readable for the given source. **Throws:**: `java.lang.UnsupportedOperationException` **See Also:**: `Property.isWriteable(S)`

---


### isWriteable

```
public abstract boolean isWriteable(S source)
```

:   Returns whether or not the `Property` is writeable for the given source.

    :   **Specified by:**: `isWriteable` in class `Property<S,V>`
    :   **Parameters:**: `source` - the source object on which to operate **Returns:**: whether or not the `Property` is writeable for the given source. **Throws:**: `java.lang.UnsupportedOperationException` **See Also:**: `Property.isReadable(S)`

---


### listeningStarted

```
protected void listeningStarted(S source)
```

:   Called when this `PropertyHelper` changes from having
    no listeners installed for the given source object to
    having listeners installed for the given source object. This
    is the ideal time for subclasses to install any listeners needed
    to track change on the source object.

    :   **See Also:**: `listeningStopped(S)`

---


### listeningStopped

```
protected void listeningStopped(S source)
```

:   Called when this `PropertyHelper` changes from having
    listeners installed for the given source object to
    having no listeners installed for the given source object. This
    is the ideal time for subclasses to remove any listeners that
    they've installed to track changes on the source object.

    :   **See Also:**: `listeningStopped(S)`

---


### addPropertyStateListener

```
public final void addPropertyStateListener(S source,
                                           PropertyStateListener listener)
```

:   Adds a `PropertyStateListener` to be notified when the state of the
    `Property` changes with respect to the given source. Does nothing if
    the listener is `null`. If a listener is added more than once,
    notifications are sent to that listener once for every time that it has
    been added. The ordering of listener notification is unspecified.

    :   **Specified by:**: `addPropertyStateListener` in class `Property<S,V>`
    :   **Parameters:**: `source` - the source object on which to operate: `listener` - the listener to be notified

---


### removePropertyStateListener

```
public final void removePropertyStateListener(S source,
                                              PropertyStateListener listener)
```

:   Removes a `PropertyStateListener` for the given source. Does
    nothing if the listener is `null` or is not one of those registered
    for this source object. If the listener being removed was registered more
    than once, only one occurrence of the listener is removed from the list of
    listeners. The ordering of listener notification is unspecified.

    :   **Specified by:**: `removePropertyStateListener` in class `Property<S,V>`
    :   **Parameters:**: `source` - the source object on which to operate: `listener` - the listener to be removed **See Also:**: `Property.addPropertyStateListener(S, org.jdesktop.beansbinding.PropertyStateListener)`

---


### getPropertyStateListeners

```
public final PropertyStateListener[] getPropertyStateListeners(S source)
```

:   Returns an arry containing the listeners registered for the given source.
    Order is undefined. Returns an empty array if there are no listeners.

    :   **Specified by:**: `getPropertyStateListeners` in class `Property<S,V>`
    :   **Parameters:**: `source` - the source object on which to operate **Returns:**: the set of listeners registered for the given source **See Also:**: `Property.addPropertyStateListener(S, org.jdesktop.beansbinding.PropertyStateListener)`

---


### firePropertyStateChange

```
protected final void firePropertyStateChange(PropertyStateEvent pse)
```

:   Notify listeners that the state of this property has changed, as
    characterized by the given `PropertyStateEvent`. If this
    `PropertyHelper` is managing listeners for multiple sources, only
    the listeners associated with the object returned by the
    `PropertyStateEvent's getSourceObject()` method are notified.

    :   **Parameters:**: `pse` - the `PropertyStateEvent` characterizing the state change

---


### isListening

```
public final boolean isListening(S source)
```

:   Returns whether or not there are any `PropertyStateListeners`
    installed for the given source object.

    :   **Parameters:**: `source` - the source object of interest **Returns:**: whether or not there are any `PropertyStateListeners` installed for the given source object


---


|  |  |  |
| --- | --- | --- |
| **Overview** \| **Package** \| **Class** \| **Use** \| **Tree** \| **Deprecated** \| **Index** \| **Help** | |  |
| **PREV CLASS**   **NEXT CLASS** | **FRAMES**    **NO FRAMES**     **All Classes** |  |
| SUMMARY: NESTED \| FIELD \| CONSTR \| METHOD | DETAIL: FIELD \| CONSTR \| METHOD |  |


---
